# Cylindromatosis Lysine 63 Deubiquitinase (CYLD) Regulates NF-kB Signaling Pathway and Modulates Fibroblast and Endothelial Cells Recruitment in Nasopharyngeal Carcinoma

**DOI:** 10.3390/cancers12071924

**Published:** 2020-07-16

**Authors:** Mingdan Deng, Wei Dai, Valen Zhuoyou Yu, Lihua Tao, Maria Li Lung

**Affiliations:** Department of Clinical Oncology, University of Hong Kong, 21 Sassoon Road, Pokfulam, Hong Kong (SAR), China; u3003939@connect.hku.hk (M.D.); weidai2@hku.hk (W.D.); zvyu@hku.hk (V.Z.Y.); taolihua@hku.hk (L.T.)

**Keywords:** NF-kB, cylindromatosis lysine 63 deubiquitinase (*CYLD*), nasopharyngeal carcinoma (NPC), tumor microenvironment

## Abstract

Nasopharyngeal carcinoma (NPC) is a malignant epithelial carcinoma of the nasopharynx. Cylindromatosis lysine 63 deubiquitinase (*CYLD*), a NF-kB inhibitor, was reported as one of the top mutated candidate genes in NPC. NF-kB is an inducible transcription factor, contributing to cancer via regulating inflammation, angiogenesis, cell proliferation, and metastasis. In this study, the impact of *CYLD* on regulating the NF-kB signaling pathway and its contribution to NPC development was studied using in vitro and in vivo functional assays, together with single cell RNA sequencing to understand the NPC tumor microenvironment. *CYLD* was downregulated in NPC clinical specimens and multiple cell lines. Functional assays revealed *CYLD* inhibits NPC cell proliferation and migration in vitro and suppresses NPC tumorigenicity and metastasis in vivo by negatively regulating the NF-kB signaling pathway. Additionally, *CYLD* was able to inhibit fibroblast and endothelial stromal cell infiltration into the NPC tumor microenvironment. These findings suggest that *CYLD* inhibits NPC development and provides strong evidence supporting a role for *CYLD* inhibiting fibroblast and endothelial stromal cell infiltration into NPC via suppressing the NF-kB pathway.

## 1. Introduction

Nasopharyngeal carcinoma (NPC) is a malignant epithelial cancer arising in the nasopharynx with a distinct gender, geographical, and racial distribution worldwide. It is rare among Caucasians with a frequency of less than 1 case per 100,000 and is dramatically more common in Southeast Asia, Southern China, Singapore, North Africa, and in the Alaskan Eskimos of the USA [1,2]. Hong Kong-wide, it ranks tenth in cancer mortality. The pathogenesis of NPC is attributed to three main etiological factors: host genetics, Epstein–Barr Virus (EBV) infection, and environmental factors.

Cylindromatosis lysine 63 deubiquitinase (*CYLD*), a tumor suppressor gene on chromosome 16q12–13, was first identified as a gene mutated in familial cylindromatosis [2,3]. It encodes a CYLD protein containing three cytoskeletal-associated protein-glycine-conserved (CAP-Gly) repeats and one zinc finger-like B-box motif (USP) domain. Its deficiency occurs in different types of disease, including familial cylindroma, Brook–Spiegler syndrome, head and neck, cervical, colon, and prostate cancers [4]. Our previous whole-exome sequencing (WES) and targeted sequencing study identified *CYLD,* a NF-kB inhibitor, as one of the top mutated NPC genes [5]. CYLD, a deubiquitination (DUB) enzyme, acts as a negative regulator in NF-kB signaling by deubiquitination of its upstream signaling actors, including TRAF2, TRAF6, RIP1, and NEMO [6,7,8,9]. NF-kB plays a critical role in NPC via regulating a variety of functions, including inflammation, immune response, cell proliferation and survival [10,11]. Dysregulation of NF-kB is considered a critical component of NPC tumorigenesis [12]. NPC tumors are infamous for having a heavy lymphocytic infiltration [13,14]. This tumor microenvironment (TME) plays a vital role in NPC tumor initiation and development. However, to-date, there is only limited research focusing on TME contributions in NPC.

Although there are a number of studies associated with *CYLD* function in different cancers, its function has yet to be more extensively explored in NPC. Deciphering its roles in the molecular signaling pathways will improve our understanding of the molecular genetic basis of NPC and identify possible gene targets for therapeutic treatment. *CYLD* is often inactivated by genetic alterations or downregulated in NPC clinical specimens. The in vivo and in vitro assays suggest *CYLD*, as a negative regulator of the NF-kB signaling pathway in NPC, affects tumor growth, metastasis and the TME composition. It holds promise as a potential molecular therapeutic target for NPC patients.

## 2. Results

### 2.1. Integrative Genomic Analysis Reveals Multiple Mechanisms Leading to CYLD Inactivation and Down-Regulation in NPC

**Figure 1:**
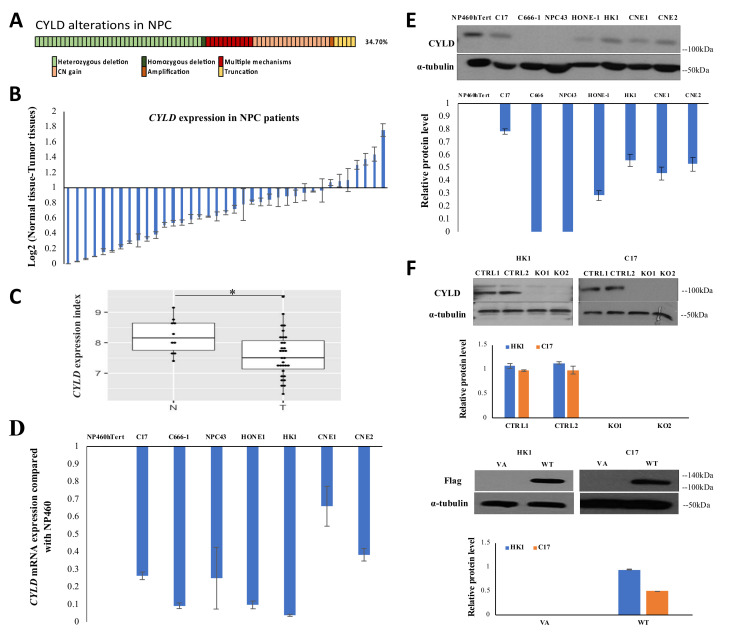
*CYLD* expression in NPC cell lines and clinical patients. (**A**) Integrative genomic analysis identified *CYLD* somatic alterations in 34.7% of cases. (**B**) A total of 37 pairs of NPC normal/tumor tissues were used for real-time quantitative polymerase chain reaction (qRT-PCR) analysis. *CYLD* mRNA was down-regulated in 30 of 37 (81%) cases. The data shown are represented as mean ± standard deviation (SD); *n* = 4. (**C**) Publicly available microarray data (GSE12452) show that *CYLD* was significantly downregulated in NPC tumors (31 cases) compared to normal tissues (10 cases). N: normal; T: tumor. *: *p*-Value < 0.05. (**D**) The qRT-PCR result shows *CYLD* mRNA expression was downregulated in C17, C666-1, NPC43, HONE-1, HK1, CNE1, and CNE2 compared to NP460hTert. (**E**) The western blot result shows that CYLD protein was downregulated in seven cell lines compared to NP460hTert. The α-tubulin was used as the loading control. Quantification by densitometry is shown in the bar graph. (**F**) Two pairs of sgRNA-targeted *CYLD* were utilized to create a functional knockout of *CYLD* and two pairs of sgRNA-nontarget were utilized as the control in HK1 and C17, as verified by western blots. The overexpression effect of FLAG-tag *CYLD*/VA plasmid infection of HK1 and C17 cell lines was verified by western blot with quantification by densitometry, as shown in the bar graph. CTRL1: nontarget control1; CTRL2: nontarget control2; KO1: *CYLD*-targeted knockout1; KO2: *CYLD*-targeted knockout2. VA: overexpression vector-alone; WT: overexpression *CYLD* wild-type. The whole western blot for Figure 1E is shown in the Figure S10. The whole western blots for Figure 1F are shown in the Figure S11.

We integrated the WES and whole genome sequencing (WGS) data from three genomic studies [5,15,16] and identified somatic changes at *CYLD* in 34.7% of the cases (Figure 1A). The *CYLD* alterations include deletions, truncations, and tandem duplication; eleven cases have more than one mechanism involved (Table S1). All the *CYLD* mutations identified in the analysis are listed in Table S2. These results suggest multiple mechanisms lead to *CYLD* inactivation or down-regulation that potentially impair its biological function in NPC. In addition to frequently mutated *CYLD*, this gene is down-regulated in 37 panels of NPC tumors compared to the paired normal tissues (Figure 1B); it is also down-regulated in 31 NPC tumors compared to 10 normal tissues in a publicly available microarray analysis (Figure 1C). To further verify the significance of *CYLD* in NPC, its protein and mRNA expression levels were assessed in seven NPC cell lines (C17, C666-1, NPC43, HONE-1, HK1, CNE1, CNE2), compared to the immortalized nasopharyngeal epithelial NP460hTert cell. CYLD protein and mRNA were both downregulated in all NPC cell lines compared with NP460hTert (Figure 1D,E). Noticeably, loss of CYLD was found in C666-1 and NPC43, which is potentially relevant to the truncation mutation and deletion, respectively (Table S3). Thus, the extensive *CYLD* downregulation and mutations observed suggest its potentially important role in NPC tumorigenesis.

### 2.2. CYLD Expression Level Affects NPC Tumor Growth In Vivo and Cell Proliferation In Vitro

**Figure 2:**
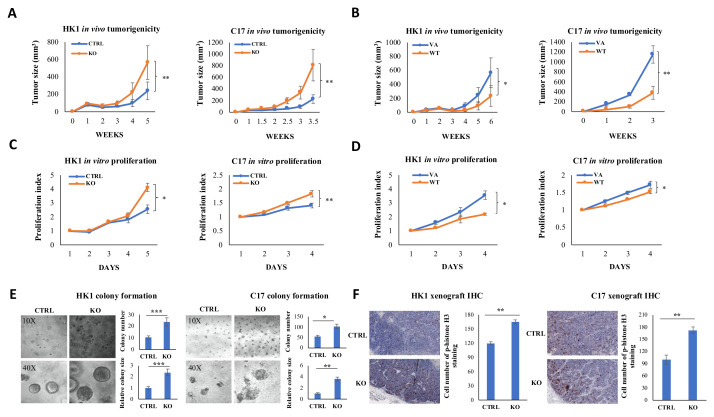
*CYLD* inhibits NPC tumorigenicity in vivo and cell proliferation in vitro. (**A**) Nude mice subcutaneous injection was performed to assess *CYLD* effects on tumorigenicity in vivo. After *CYLD* knockout, both HK1 and C17 show significantly faster tumor growth compared to the control. *n* = 12. (**B**) With *CYLD* over-expression, both HK1 and C17 show significantly slower tumor growth than for the VA. *n* = 12. (**C**) *CYLD* knockout increases cell proliferation. (**D**) *CYLD* overexpression suppresses cell proliferation in HK1 and C17. (**E**) HK1 and C17 cells with *CYLD* knockout form more colonies with larger sizes in the 3D colony formation assay. Representative images are shown under 10× and 40× magnification. (**F**) Immunohistochemical (IHC) staining of HK1 and C17 xenografts shows more cells in mitosis in the *CYLD* knockout group. The quantitative data are shown in the bar graphs. Scale bar = 1000 μm. The data are represented as mean ± standard deviation (SD); *n* = 3. CTRL: nontarget control; KO: *CYLD*-targeted knockout. *: *p*-Value < 0.05; **: *p*-Value < 0.01; ***: *p*-Value < 0.001.

To examine *CYLD* function in vivo, *CYLD* was knocked out and over-expressed in two NPC cell lines, HK1 and C17, followed by Western blot to confirm knockout and over-expression effects (Figure 1F). The engineered cells were injected subcutaneously into athymic mice to examine tumorigenicity. In both HK1 and C17, *CYLD* knockout enhances in vivo tumor growth compared to the non-targeted control, while *CYLD* overexpression suppresses tumor growth compared to vector-alone (VA) (Figure 2A,B). *In vitro* cell proliferation was measured and the result shows that *CYLD* knockout causes cell proliferation enhancement and *CYLD* overexpression causes cell proliferation suppression (Figure 2C,D). The cell cycle arrest assay shows HK1 cells with *CYLD* knockout transit faster from S phase to G2 phase (Figure S1). In addition, we further examined cell growth by performing the 3D colony formation assay. Both HK1 and C17 cells with *CYLD* knockout formed significantly more colonies with larger sizes compared to the control (Figure 2E). Our immunohistochemical (IHC) staining of p-histone H3 confirmed the xenografts with *CYLD* knockout contain greater numbers of mitotic cells than in controls (Figure 2F). These results collectively indicate a functional role of *CYLD* in suppressing NPC tumorigenicity in vivo and cell proliferation *in vitro.*

### 2.3. CYLD Expression Level Affects NPC Metastasis In Vivo and Migration In Vitro

**Figure 3:**
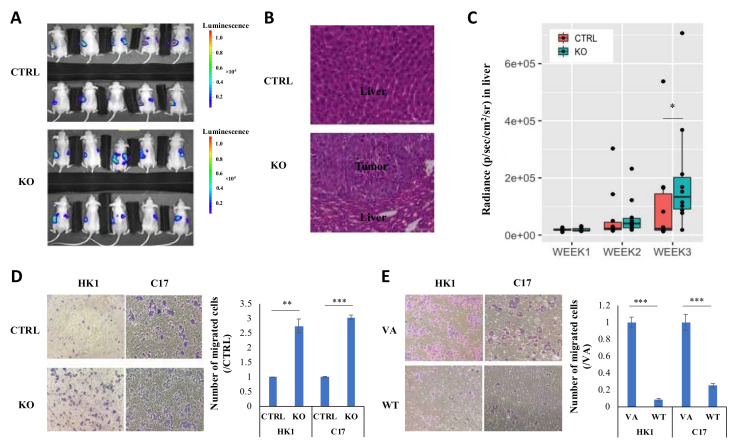
*CYLD* inhibits NPC metastasis in vivo and migration in vitro. (**A**) HONE-1-luc cell line with *CYLD* knockout/control were intrasplenically injected into nude mice (ten mice per group). The third-week bioluminescent signal was assessed by the IVIS Spectrum system as shown. (**B**) H and E staining was performed to confirm the tumor metastasis to liver. One liver from the control group and one liver from the knockout group under 20× magnification are shown. Scale bar = 100 μm. (**C**) Bioluminescence signals from control and *CYLD* knockout were quantified weekly. Radiance of mice in both groups are represented in box plots. RStudio was used to draw the box plot. The signal in the knockout group is significantly higher than in the control group. (**D**) Boyden chamber migration assay was performed in both HK1 and C17 cells with *CYLD* knockout/control. (**E**) *CYLD* overexpression/VA. Representative images under 10× magnification and the quantitative bar graphs show the normalized migrated cell numbers. The data shown are represented as the mean ± standard deviation (SD). CTRL: nontarget control; KO: *CYLD*-targeted knockout; VA: overexpression vector-alone; WT: overexpression *CYLD* wild-type. *: *p*-Value < 0.05; **: *p*-Value < 0.01; ***: *p*-Value < 0.001.

Previous reports indicate that functional inactivation of *CYLD* promotes the metastasis in squamous cell carcinomas [17]. However, the role of *CYLD* on metastasis has not been well-studied in NPC. To investigate this effect, HONE-1-luciferase labeled (HONE-1-luc) cells were intrasplenically injected into the nude mouse model. By the third week, the *CYLD* knockout group has more mice with detectable signals from liver metastasis than controls (Figure 3A). The body weight of each group was measured and recorded in Figure S2. Although the mean of the body weights of the *CYLD* knockout group is lower than the control, because of the severe liver metastasis, there is no significant difference between these two groups. At necropsy, metastatic lesions in the liver were confirmed by hematoxylin and eosin (H and E) staining (Figure 3B). After *CYLD* knockout, more mice had liver metastasis than the control (Figure 3A) and in particular, the quantitative analysis revealed the bioluminescence signal from the liver is much higher in the *CYLD* knockout group than the control group by week 3 (Figure 3C). These results support a role for *CYLD* suppressing NPC growth and metastasis in vivo. The in vitro chamber migration and wound healing assays show that *CYLD* dramatically suppresses cell migration in both HK1 and C17 (Figure 3D,E, Figure S3). The data are consistent with *CYLD* acting as a tumor suppressor gene to inhibit cell migration in vitro and metastasis *in vivo.*

### 2.4. CYLD Suppresses NF-kB Signaling Pathway via Inhibiting p65 Nuclear Translocation

**Figure 4:**
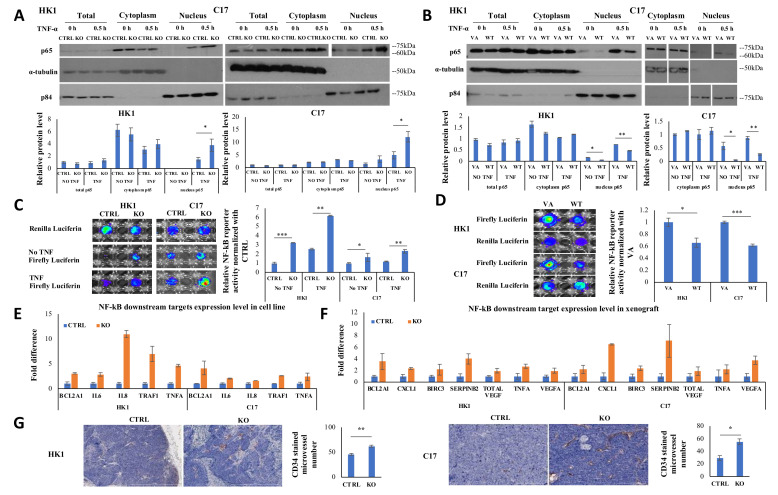
*CYLD* acts as a negative regulator of the NF-kB pathway in NPC. (**A**) HK1 and C17 with *CYLD* knockout/control were treated with 30 ng/mL TNF-α for 0.5 hour prior to subcellular fractionation and Western blot analysis to detect p65 nucleus translocation. The quantification by densitometry is shown in the bar graphs. Without TNF-α stimulation, most p65 localizes to the cytoplasm. After 0.5 hour of stimulation, more p65 was translocated into the nucleus in both HK1 and C17 with *CYLD* knockout compared to control. The p84 and α-tubulin were used as loading controls. (**B**) HK1 and C17 overexpressing *CYLD*/VA were treated with 30 ng/mL TNF-α for 0.5 hour and then subjected to subcellular fractionation and Western blot with quantification by densitometry shown in the bar graphs. Consistently, less p65 was translocated into nucleus in the cells with *CYLD* overexpression compared to the VA. (**C**,**D**) the NF-kB-specific reporter assay was used to examine the NF-kB signals in HK1 and C17. Renilla luciferase serves as a normalization control. Firefly luciferase indicates NF-kB reporter signal. Quantification of the bioluminescent signal is shown in the bar graph. (**E**) NF-kB downstream targets were screened by qRT-PCR in both HK1 and C17 cells with *CYLD* knockout/control. Several genes with over two-fold differences in both HK1 and C17 are shown. (**F**) NF-kB downstream targets were screened by qRT-PCR in both HK1 and C17 xenografts with *CYLD* knockout/control. More NF-kB downstream targets were upregulated. (**G**) IHC staining of the HK1 and C17 xenografts shows more microvessel formation (Cd34-positive) after *CYLD* knockout, as compared to control. ImageJ was used to count microvessel numbers. Scale bar = 1000 μm. The data shown are represented as mean ± standard deviation (SD); *n* = 3. CTRL: nontarget control; KO: *CYLD*-targeted knockout; VA: overexpression vector-alone; WT: overexpression *CYLD* wild-type. *: *p*-Value < 0.05; **: *p*-Value < 0.01; ***: *p*-Value < 0.001. The whole western blots for Figure 4A are shown in the Figure S12. The whole western blots for Figure 4B are shown in the Figure S13.

*CYLD* acts as an inhibitor of the NF-kB signaling pathway via the canonical pathway [8,18,19]. To assess the potential function of *CYLD* on NF-kB, its effect on p65 protein expression and localization was examined in HK1 and C17. Figure 4A,B show the total p65 expression remains the same after *CYLD* knockout or overexpression; in contrast, after 0.5 hour of TNF-α stimulation, more p65 translocates into the nucleus after *CYLD* knockout compared to the control and less p65 is detected in the nucleus after *CYLD* overexpression compared to the vector alone (VA). Meanwhile, the immunofluorescence (IF) staining of p65 in HK1 and C17 cells (Figure S4) consistently indicates the cytoplasmic p65 translocated into the nucleus after *CYLD* knockout. These findings were verified by in vivo p65 staining in the xenografts, which show more cells with p65 nuclear staining after *CYLD* knockout compared with control (Figure S5), suggesting *CYLD* expression contributes to inhibition of p65 nuclear translocation from the cytoplasm.

### 2.5. CYLD Expression Affects NF-kB Transcriptional Function and CYLD Knockout Increases Angiogenesis

Consequences of altered p65 translocation into the nucleus was examined using an NF-kB-specific reporter assay. After normalization of the renilla luciferase signal to the firefly luciferase signal, *CYLD* knockout results in a significant increase in the normalized luciferase activity of the vector containing the p65-binding site compared with the control (Figure 4C), while CYLD overexpression induces lower luciferase activity compared with the VA (Figure 4D). The consequence of this was further examined for NF-kB downstream target expression levels in both cell line level and xenograft by qRT-PCR. The results for all the NF-kB downstream genes we screened in cell lines and xenografts are shown in Figure S6. A number of genes (*BCL2A1, IL6, IL8, TRAF1, TNFA*) were upregulated over two-fold, after *CYLD* knockout in both HK1 and C17 cells (Figure 4E). With the regulation of the tumor microenvironment, more genes were upregulated with over two-fold differences in both HK1 and C17 xenografts after *CYLD* knockout, including *BCL2A1, CXCL1, BIRC3, SERPINB2,* total *VEGF, TNFA,* and *VEGFA* (Figure 4F). As NF-kB signaling is a well-known pathway for promoting tumor angiogenesis [20] and results showed increased total *VEGF* and *VEGFA* expression after *CYLD* knockout, tumor microvessel formation was investigated by IHC staining of Cd34 in xenografts. The *CYLD* knockout HK1 and C17 xenografts formed significantly more microvessels than controls (Figure 4G). These data further confirmed knockout of *CYLD* induces NF-kB hyperactivity.

### 2.6. Single Cell Analysis Shows CYLD Suppresses Specific Stromal Cell Infiltration in the TME

**Figure 5:**
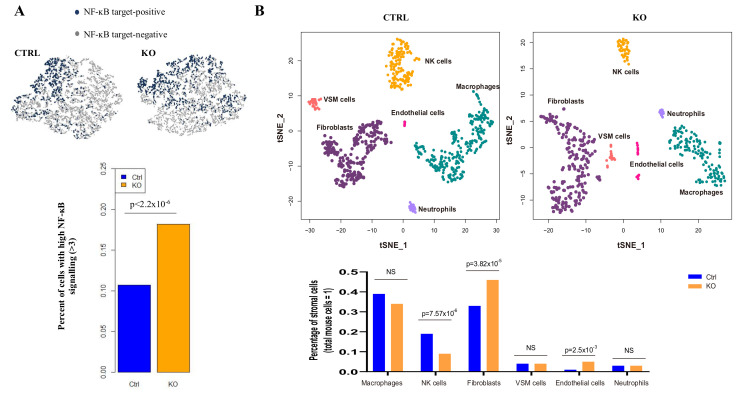
*CYLD* plays critical roles in stromal cell infiltration in TME. (**A**) Mouse xenografts established from HK1 cells transduced with control (*n* = 2) and *CYLD* knockout (*n* = 2) were used for single-cell RNA-Seq analysis. The panel of NF-kB downstream genes mediated by *CYLD* was set as an indicator of high NF-kB activity in human cancer cells. These genes include *BCL2A1, CXCL10, MMP9, SERPINB2*, total *VEGF, TNFA, VEGFA, SOD2, VCAM, CXCL1*, and *EGFR*. The clusters show single cells with NF-kB target-positive (blue color)/target-negative (grey color). The bar graph shows significantly more cells in the *CYLD* knockout xenograft with high NF-kB signaling than the control (*p*-Value < 2.2 × 10^−6^). (**B**) After quantification analysis from the single-cell RNA sequencing data, six clusters are identified, indicating fibroblasts, endothelial cells, natural killer cells, neutrophils, vascular smooth muscle (VSM) cells, and macrophages. Quantitative analysis of the percentage of each of these stromal cells compared to total mouse cells is shown in the bar graph. The *p*-Value estimated from a chi-square test for each category of cells is presented. A *p*-Value < 0.05 shows a significant difference between the two groups. NS: not statistically significant.

*CYLD* acts as a negative regulator in the NF-kB signaling pathway in NPC. Knockout of *CYLD* results in hyperactive NF-kB activity, which may regulate the host immune response, resulting in altered stromal cell composition to support tumor growth. A single-cell RNA sequencing approach was used to investigate the impact of loss of *CYLD* on the TME in HK1 xenografts. The genes from mouse stromal cells and human cancer cells were analyzed. qRT-PCR results showed the *CYLD* knockout induces upregulation of a panel of NF-kB downstream genes (Figure S7); we examined the expression of these CYLD-targeted genes in the single-cell data and discovered that there are almost twice as many cells with hyperactivated NF-kB activity in the cancer cells with the *CYLD* knockout compared to the control (Figure 5A).

These results indicate that *CYLD* knockout resulted in hyperactive NF-kB signaling, which could affect the stromal TME. Figure 5B shows the single-cell RNA sequencing data classified six major mouse stromal components of non-epithelial cells, including macrophages, fibroblasts, endothelial cells, neutrophils, vascular smooth muscle (VSM) cells, and natural killer (NK) cells. Quantitative analysis of the single-cell data showed that there are significantly increased numbers of mouse fibroblasts and endothelial cells and decreased NK cell infiltration in *CYLD* knockout xenograft compared to the control xenograft (Figure 5B). As previously reported, cancer-associated fibroblasts (CAFs) are activated to promote macrophage recruitment, angiogenesis, and tumor growth in the NF-kB cascade [21]. Therefore, *CYLD* expression could alter CAF infiltration in TME to regulate tumor growth via the NF-kB pathway.

### 2.7. CYLD Suppresses Fibroblast and Endothelial Cell Infiltration in NPC TME

**Figure 6:**
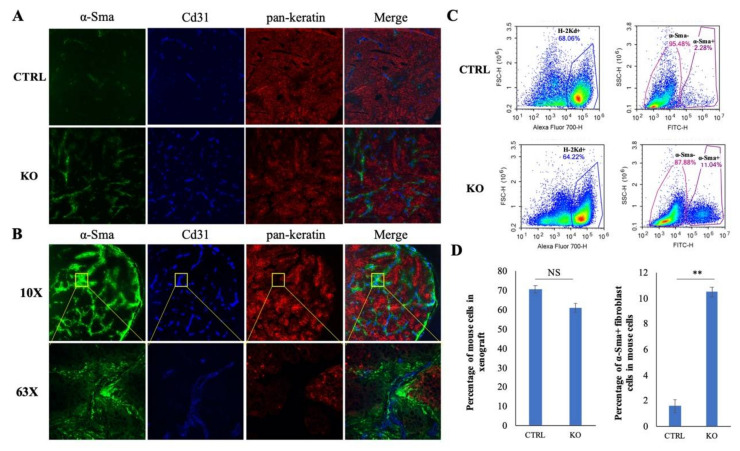
*CYLD* inhibits fibroblast and endothelial cell infiltration to the TME. (**A**) After three weeks of subcutaneous growth in nude mice, xenografts were excised (*n* = 3 for CYLD control and *n* = 3 for CYLD KO), followed by IF staining. Three images were taken under a 10× objective lens for each tumor. More fibroblast (α-Sma-positive) and endothelial cells (Cd31-positive) were detected in the xenograft with *CYLD* knockout compared with the control. (**B**) Images under a 10× and 63× objective lens show fibroblasts localized adjacent to endothelial cells. The images under a 63× objective lens show cancer cells adjacent to fibroblast and endothelial cells have strong α-Sma-positive staining. (**C**) Flow cytometry analysis of single cells dissociated from xenograft shows significantly more fibroblast cells in *CYLD* knockout xenograft compared with control. (**D**) Quantification of the flow cytometry data are plotted in the bar graph on the left, indicating the percentage of mouse cell infiltration into the tumor, and the bar graph on the right indicating the percentage of fibroblast cells out of total mouse cells is significantly higher in the *CYLD* knockout. The data shown are represented as mean ± standard deviation (SD). CTRL: nontarget control; KO: *CYLD*-targeted knockout. NS: not statistically significant. **: *p*-Value < 0.01.

In support of the single-cell data, IF staining of tumor specimens using α-Sma and Cd31 antibodies to classify fibroblast and endothelial cells, respectively, and pan-keratin to detect the cancer cells was performed. The IF staining shows that the *CYLD* knockout xenograft has a much greater proportion of fibroblasts and endothelial cells than the control (Figure 6A), in concordance with the sequencing data (Figure 5B). Furthermore, the staining result shows the α-Sma-positive fibroblasts located adjacent to the Cd31-positive vasculature (Figure 6B), as was observed directly in clinical NPC specimens [22], suggesting CAFs may enhance neo-angiogenesis in NPC. Moreover, the cancer cells adjacent to fibroblasts and vasculature show high α-Sma staining in the *CYLD* knockout xenograft. As previously reported, cancer cells with high α-Sma expression show enhanced metastasis and poor prognosis in lung adenocarcinoma [23] and higher expression of α-Sma was detected in malignant ovarian epithelial neoplasms than in the benign tumors [24], suggesting cancer cells with high α-Sma expression may be more aggressive. Furthermore, flow cytometry quantitative analysis of single cells dissociated from the xenografts demonstrated an increase of around five-fold fibroblasts, after *CYLD* knockout (Figure 6C,D). Thus, *CYLD* appears to inhibit NPC tumor recruitment of stromal cells, including fibroblasts and endothelial cells into the TME.

### 2.8. CYLD Truncation Mutations (S323X, S371X) Abolish Wild-Type (WT) Function as a NF-kB Inhibitor

**Figure 7:**
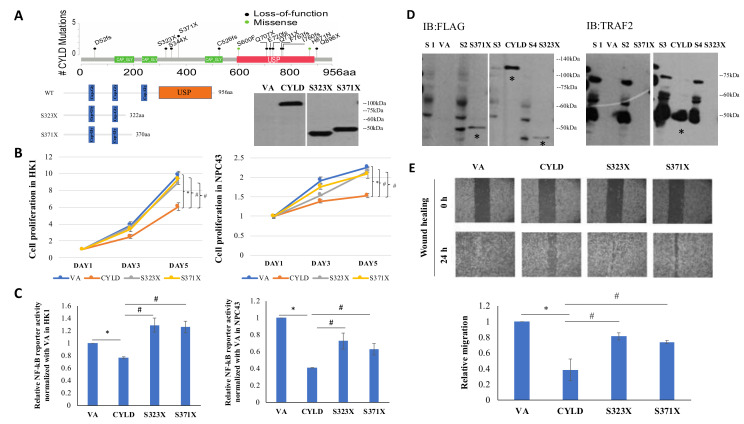
Functional study of *CYLD* somatic mutants S323X and S371X. (**A**) Schematics of *CYLD* (NM_015247) mutations detected from three NPC tumor genomic studies [5,15,16]. A hot spot *CYLD* mutation S371X was identified. CAP-GLY, cytoskeleton-associated proteins-gly-rich domain; USP, ubiquitin-specific protease domain for top schematic. The constructed FLAG-tagged WT and S323X and S371X mutation plasmids were transfected in HEK293-FT to verify mutation expression by western blot. (**B**) The HK1 and NPC43 cells with S323X and S371X overexpression proliferate significantly faster than with CYLD wild-type overexpression. (**C**) NF-kB activity in NPC cells with VA, CYLD, S323X, S371X transduction was assessed with a p65 signaling reporter assay. Significantly higher p65 signaling is seen in NPC43 and HK1 with S323X and S371X overexpression. (**D**) Co-IP assay was performed to check protein interaction between S323X, S371X, and the CYLD target, TRAF2, in HEK293-FT cells. FLAG-tagged beads were used for immunoprecipitation (IP). The FLAG antibody was used to examine the CYLD pull-down. *: band expected. S1: total lysis of cells with VA; S2: total lysis of cells with S371X; S3: total lysis of cells with WT; S4: total lysis of cells with S323X. (**E**) Wound healing assay was performed to evaluate cell migration. The width of the wound was recorded under 4× magnification at 0 and 24 hours after the wound scratch. The data represent three independent experiments to show relative migration rates. VA: overexpression vector-alone; WT: overexpression CYLD wild-type. *: *p*-Value < 0.05 compared to VA, #: *p*-Value < 0.05 compared to WT. The whole western blot for Figure 7A is shown in the Figure S14. The whole western blots for Figure 7D are shown in the Figure S15.

Our previous WES and targeted sequencing identified five *CYLD* mutations in NPC patients [5]. We have now integrated *CYLD* mutations detected in three NPC genomics studies [5,15,16], as shown in Figure 7A. The S371X was found to be a hot spot mutation. FLAG-tagged mutation plasmids were constructed by a site-directed mutagenesis system for S323X and S371X, as we had detected in our previous NPC WES study [5] and their protein expression in cells was confirmed by Western blot (Figure 7A). The *CYLD* truncation mutations, S323X and S371X, lost most of the *CYLD* functional domain (Cap-Gly and USP domain). As shown in Figure 7B, loss of this important region was crucial for *CYLD* WT function to suppress cell proliferation in both HK1 and NPC43. Furthermore, the NF-kB reporter assay was used to verify the function of S323X and S371X in NPC. Results indicate *CYLD* overexpression results in a significant decrease in the normalized luciferase activity of the vector containing the p65-binding site compared with the VA. Moreover, both S323X and S371X contribute to significantly higher normalized luciferase activity compared with *CYLD* overexpression (Figure 7C). TRAF2 is one of canonical NF-kB signaling upstream regulators. *CYLD* can inhibit NF-kB signaling via deubiquitinating TRAF2 [18]. A Co-IP assay confirms a mechanism of S323X and S371X failure to suppress NF-kB activity by losing interaction with the NF-kB positive regulator, TRAF2 (Figure 7D). Moreover, the wound healing assay shows that *CYLD* WT inhibits cell migration, while S323X and S371X showed significant differences with the WT (Figure 7E). These data suggest the *CYLD* truncation mutants lacking the USP domain, S323X, and S371X, lose *CYLD* function as a tumor suppressor gene that impacts and inhibits NF-kB signaling.

## 3. Discussion

The tumor-suppressing function of *CYLD* has been detected in many cancers, including breast, oropharynx, colon, and hepatocellular carcinomas [25,26,27,28]. Our previous WES data first showed that *CYLD* was one of the top mutated genes (2.9%, 4 out of 135) in NPC patients [5]. A later NPC study reported *CYLD* was somatically mutated in up to 18.6% with non-synonymous mutation, translocation homozygous deletion, and tandem duplication in NPC patients [16]. We further integrated genomic analyses to identify somatic alterations on *CYLD* in 34.7% cases. These sequencing data suggest that mutations in *CYLD* are important contributors to NPC development. Microarray from publicly available data and our own qRT-PCR test of 37 pairs of NPC patient tissues show low *CYLD* expression in NPC patients.

Our knockout and overexpression results consistently show the *CYLD* expression inhibits NPC cell proliferation and delays cell transition from early S to G2 phase in the cell cycle in vitro. Both colony number and colony size increased in *CYLD* knockout cells in the 3D colony formation assay. In concordance with the in vitro assays, the *CYLD* knockout significantly enhanced tumorigenicity and *CYLD* overexpression significantly inhibit tumorigenicity in the mouse model. Collectively, this confirms that *CYLD* mediates cell proliferation and plays an important role to suppress NPC tumorigenicity.

NPC is most commonly observed to metastasize to regional lymph nodes [13] and rather more uncommonly metastasizes to distant sites such as the bone, lung, and liver [29,30,31]. High frequency of NPC metastasis increases the difficulties in successful treatment for patients and is a significant challenge for clinicians [32]. *CYLD* expression in NPC dramatically affects the cell migration ability and metastasis potential.

NF-kB is constitutively activated in NPC [33] via genetic alterations or LMP1 overexpression; *CYLD* is a key inhibitor of this pathway. We show *CYLD* inhibits p65 nuclear translocation, transcriptional activity, and *CYLD* knockout results in up-regulation of NF-kB downstream genes, including cytokines, which could induce TME alterations. With the TME regulation, we observed stronger NF-kB signaling *in vivo.* Furthermore, we detected more microvessel formation in the *CYLD* knockout xenograft. Therefore, *CYLD* plays a vital role in regulating NPC via mediating three cancer hallmarks, including proliferation, angiogenesis, and metastasis.

NPC is characterized as a stromal-enriched malignancy with heavy immune-cell infiltration, indicating the importance of the TME in NPC tumorigenesis. CYLD inhibitory roles in the NF-kB pathway may serve as a link between cancer cells and TME. NF-kB mediates a crosstalk between inflammation and cancer in multiple aspects. Increased NF-kB signaling activity results in accumulation of pro-inflammatory cytokines from the tumor site, contributing to the pro-tumorigenic microenvironment. The chronic inflammatory microenvironment from other sites may induce immunosuppression and favor tumor escape from immunosurveillance [34]. Using unbiased single-cell RNA sequencing to identify the cell composition in TME, we found increased fibroblasts and endothelial cells and decreased NK cells after *CYLD* knockout, suggesting *CYLD* has a critical role in regulating stromal infiltration. The NK cell is a cytotoxic effector in the human immune system defense against infection and tumor development. NPC patients with high-density NK cell infiltration show better OS and PFS, serving as a potential indicator for predicting recurrence and distant metastasis [35]. Our data show decreased NK cell infiltration after *CYLD* knockout, indicating *CYLD* may play a critical role in controlling NPC development and could be a potential therapeutic target. In addition, our findings confirmed significant fibroblasts and endothelial cell alterations, indicating CYLD could regulate TME composition by inhibiting the NF-kB signaling pathway. CYLD also plays critical roles in the Wnt signaling pathway, which was reported to closely regulate TME [36,37]. A previous study reported CYLD inhibits lung fibrosis development via the TGF-β signaling pathway [38]. Our study now shows more cancer cells with hyperactivity in NF-kB signaling after CYLD knockout (11% in the control group, 18% in the knockout group, *p*-Value < 2.2×10^−6^; Figure 5A) in the single-cell RNA sequencing analysis of the two groups of mouse xenografts. While cell numbers with a high expression level of Wnt signaling-specific downstream target genes show no differences between the control (27.5%) and CYLD knockout (27.4%), TGF-β-specific downstream targets also showed no significant differences between control (8%) and knockout (10%) from the single-cell data, as shown in Figure S8. Therefore, the data suggest the CYLD knockout involves the NF-kB pathway and subsequent TME regulation instead of Wnt and TGF-β signaling pathways. NF-kB is a link between host and microenvironment and targeting the NF-kB pathway for therapy has been proposed for years [39,40]. However, the targeted therapy effect for NPC is still unsatisfactory. Targeting both NF-kB and TME to control tumor growth would be a promising direction for NPC patient management.

In this study, we investigated *CYLD* function on mediating TME components in an athymic mouse model. Xenograft models using athymic nude mice have been utilized in analyzing TME in comparison with patient tissues. Fibroblast and endothelial cells infiltration were detected to be adjacent in both nude mouse xenografts [41] and NPC patient tumor tissues [22] studies. In addition to NPC, nude mice were also used to study stromal cells in hepatocellular carcinoma [42], prostate cancer [43], and pancreatic ductal adenocarcinoma [44]. All these data suggest that xenografted tumors implanted onto athymic mice represent a suitable TME model. However, there are limitations of using the athymic mouse model, which lack T cells, for these analyses. Despite such limitations, significant insight on other intact immune components including the NK cells, neutrophils, dendritic cells, and the B cells may still be obtained [45]. In the future, immunocompetent mouse models such as transgenic/syngeneic models and humanized mouse models would be more ideal systems to utilize in mechanistic study of genes regulating TME and to evaluate the effectiveness of immunotherapy for the cancer treatment.

It has been reported that *CYLD* downregulation is an independent factor for poor prognosis in breast cancer [27] and also regulates keratinocyte differentiation and skin cancer progression [46]. Low-level *CYLD* expression was also associated with poor patient survival in oral squamous cell carcinoma [47] and inactivating *CYLD* mutations can promote skin tumor progression [48]. To date, there is still no report on *CYLD* alteration association with NPC prognosis. Our study identified two truncation mutations (S323X and one hotspot mutation S371X) associated with loss of *CYLD* wild-type function, with subsequent suppression of NF-kB signaling, cell growth, and migration. These findings highlight the need for further exploring *CYLD* correlation with NPC clinical features and treatment.

In conclusion, the findings that *CYLD* regulates three cancer hallmarks and mediates stromal cell infiltration in TME in NPC indicate it plays a major tumor-suppressive role in NPC tumorigenesis and metastasis by negatively regulating the NF-kB signaling pathway. Novel *CYLD* mutations in NPC are associated with failure to suppress NF-kB signaling and critically contribute to abolishing its function.

## 4. Materials and Methods

### 4.1. NPC Patient Specimens

A total of 37 pairs of NPC normal/tumor biopsy specimens were collected by the NPC Area of Excellence (AoE) Research Tissue Bank with written consent from patients. This study was approved by the Hospital Institutional Review Board at the University of Hong Kong (IRB# 09251).

### 4.2. Genomic Analysis in NPC

Three NPC cohorts were included in this integrative genomic analysis. The raw data were obtained from the sequence read archive (SRA) database (accession #SRP035573 and #SRA288429) and dbGAP-NHGRI (accession # phs001244.v1.p1). An additional twelve cases from our group were included (SRA database accession #SRP265671). Finally, a total of 216 NPC cases were analyzed, after removing eight cases that did not pass the quality evaluation. The raw sequencing reads were processed according to GATK Best Practices recommendations (version 3.8, BROAD INSTITUTE, Cambridge, MA, USA) [49]. The somatic SNVs and INDELs were detected by Mutect [50]. The SNVs and INDELs with at least five supporting reads and 5% allele frequency in the overlapping regions were included in the analysis. The somatic copy number alterations (SCNAs) were detected by ADTEx using the matched normal-tumor pair, as we previously described [51].

### 4.3. Cell Lines

Nine cell lines (NP460hTert, C17, C666-1, NPC43, HONE-1, HK1, CNE1, CNE2, and HEK293-FT) were used in this study. HONE-1, HK1, CNE1, CNE2, and HEK293-FT were cultured in DMEM with 5% fetal bovine serum (GIBCO, Invitrogen, Carlsbad, CA, USA) and 5% newborn calf serum (GIBCO, Invitrogen, Carlsbad, CA, USA). C666-1, NPC43, and C17 were cultured as previously described [52,53,54]. The immortalized nasopharyngeal epithelial cell line, NP460hTert, was utilized as previously described [55]. All cell lines were provided by the NPC AoE Research Tissue Bank Cell Line Repository. Mycoplasma contamination was not detected in any of these cell lines.

### 4.4. Plasmids and Lentivirus Preparation and Infection

Plasmid *CYLD* tagged with FLAG was purchased from (Addgene: #22544) and cloned into pLVX-EF1a lentiviral vector. *CYLD*-targeted single guide RNAs (sgRNA1: AAAGGCCTCCAAATAGACGT; sgRNA2: TGAGACTGAATGGTAAAGAG) were designed using the Broad Institute website and subsequently cloned into the LentiCRISPR-v2 plasmid (Addgene: #52961), as well as the non-targeted sgRNA (Sequence 1: CTCTGGCTAACGGTACGCGTA; sequence 2: CGCGATCGTAATCACCCGAGT). The *CYLD* mutation plasmids were constructed by the method of site-directed mutagenesis. Primers of these mutations were designed in GeneArt Site-Directed Mutagenesis. Lentivirus preparation and infection were performed as previously described [56].

### 4.5. Real-Time Quantitative PCR

qRT-PCR was utilized to determine the gene expression level in cell lines, xenografts, and specimens as previously described [57]. In general, the reaction is carried out with FastStart Universal SYBR Green (Roche Applied Science, Mannheim, Germany) in a LightCycler. Human glyceraldehyde 3-phosphate dehydrogenase (GAPDH) was used as the endogenous control. The relative quantity of the target gene expression was calculated by the method of 2^-ΔΔCt^. The primers used in this study are shown in Table S4.

### 4.6. Tumorigenicity in Nude Mice and Intrasplenic Metastasis Assay

*In vivo* tumorigenicity assays were performed as previously described [58] in six- to eight-week-old female athymic BALB/cAnN-nu (nude mice). Briefly, 1 × 10^7^ cells of transduced HK1 and C17 were subcutaneously injected into flanks of the mice [totally 6 mice (12 sites)/group]. Tumor size was measured and recorded weekly. NPC metastasis was investigated after intrasplenic injection in nude mice [59]. A total of 1 × 10^6^ HONE-1-luc cells were injected into the spleen following laparotomy. Totally 10 mice per group were used. Every week, the mice were monitored for metastasis by detection of bioluminescent signals on the IVIS Spectrum in vivo imaging system (PerkinElmer, Norwalk, CT, USA). Validated license from the Department of Health, Hong Kong S.A.R. to perform animal experiments was approved by the Committee on the Use of Live Animals in Teaching and Research (CULATR) of The University of Hong Kong.

### 4.7. Immunohistochemistry Staining

Immunohistochemistry (IHC) staining was performed on xenografts using p-Histone H3 (1:100, Cell Signaling Technology) and Cd34 (1:100, Santa Cruz Biotechnology, Dallas, TX, USA) antibodies. Three randomly selected locations of each stained tissue (three tumors per group) were captured for imaging analysis. The p-Histone H3-positive cells and Cd34-positive microvessels were counted by Image J software. (National Institutes of Health, Bethesda, MD, USA).

### 4.8. Cell Proliferation Assay and 3D Colony Formation

The 3-[4,5-dimethylthiazol-2-yl]-2,5 diphenyl tetrazolium bromide (MTT) assay was used to assess cell proliferation as previously described [60]. The 3-dimensional (3D) colony formation assay (CFA) was carried out to test single cell ability to form a colony [61].

### 4.9. Western Blot and Antibodies

Western blot was performed using previous protocols [62]. Antibodies include FLAG epitope tag (1:1000, MERCK), CYLD (1:1000, Cell Signaling), p65 (1:1000, Cell Signaling), TRAF2 (1:1000, Cell Signaling) and p84 (1:5000, GeneTex, Irvine, CA, USA), α-tubulin (1:5000, GeneTex) were used as loading control.

### 4.10. Subcellular Fractionation

When cell confluence reached 60–80%, cells were harvested and prepared to perform cell nucleus and cytoplasm separation as described previously [63] and used to perform western blot.

### 4.11. Co-Immunoprecipitation Assay

The co-immunoprecipitation (Co-IP) assay was performed with ANTI-FLAG M2 Magnetic Beads (MERCK) according to the protocol provided. Ten µL of lysate were utilized as the control. Western blot was used to analyze the interactions.

### 4.12. Wound Healing and Chamber Migration Assay

The wound-healing assay was used as reported previously [61]. The wound width was measured by the NIS-Elements software (Nikon, Tokyo, Japan). The wound healing percentage was calculated as: (initial width-wound width at 24 h)/initial width. The chamber assay was performed as previously described [60]. Image J software was used to count the colonies.

### 4.13. NF-kB-Specific Single Plasmid Dual Reporter Assay

The dual reporter plasmid, pTRE-fluc-EF1a-Rluc plasmid includes a binding site of NF-kB and results in expression of firefly luciferase. EF1a promoter will drive Renilla luciferase and is used as a normalization control. The reporter assay was performed in C17 and HK1, as previously described [64].

### 4.14. 10× Genomics Single Cell RNA Sequencing and Data Analysis

Two pooled xenografts of the control (*n* = 2) and *CYLD* knockout (*n* = 2) were excised and diced into small pieces for dissociation. The spun-down tissue cell pellet was passed through a 100 μm filter, followed by resuspension in 400 μL dead cell removal kit (Miltenyi Biotec, Bergisch Gladbach, Germany). The viable cells were separated from dead cells after passing through an MS column according to the protocol. The living single cells were resuspended in 0.04% BSA/PBS and processed for single-cell RNA sequencing by the Center for Genomic Sciences at the LKS Faculty of Medicine, University of Hong Kong. The sequencing reads were mapped to both the human (hg19) and mouse (mm10) transcriptomes using Cell Ranger (v3.0.0) (10× GENOMICS, Pleasanton, CA, USA). The processed data were subsequently analyzed by R package *Seurat*. The cells with at least 95% of the reads mapped to mm10 or hg19 were considered as mouse or human cells, respectively. Dimensionality reduction step for visualizing the data in two dimensions was performed using t-distributed stochastic neighbor embedding (t-SNE). The Knee plots of both groups for quality control are shown in Figure S9. In order to classify mouse stromal cell identities, the clustered cells were identified through specific known cell markers as shown in Table S5.

### 4.15. Immunofluorescence Staining of Cryosected Tissue Sections

Tissue specimens of 10 μm thickness were cut, mounted on slides, and stored at −80 °C prior to immunofluorescence (IF) staining. The slides were fixed with ice-cold methanol for 10 mins, followed by PBS washing and permeabilizing in 0.2% PBS-triton for 15 mins. After PBS washing three times, the sections were blocked for 30 mins before overnight incubation with the antibodies (α-Sma, ThermoFisher SCIENTIFIC; Cd31, BioLegend; pan-keratin, Cell Signaling Technology, Danvers, MA, USA). A mounting reagent (Prolong^TM^ Glass Antifade Mountant, Invitrogen, ThermoFisher Scientific, Carlsbad, CA, USA) was used to cover the slides. The stained images were captured on a Carl Zeiss LSM 780 confocal microscope. In total, three tumors per group were used for IF staining.

### 4.16. Flow Cytometry of Single Cells Dissociated from Xenograft

The xenograft tissue was dissociated and passed through a 100 μm filter. The collected cells were fixed in 2% paraformaldehyde for 10 mins and washed thrice in permeabilization buffer. The cells were incubated with primary antibodies (mouse cell-specific antibody, H-2Kd conjugated with Alexa Fluor 700, Miltenyi Biotec, Bergisch Gladbach, Germany and fibroblast-specific antibody, α-Sma with Alexa Fluor 488, ThermoFisher SCIENTIFIC, Rockford, MA. USA) for 1 hour, followed by three washes. The stained cells were resuspended in permeabilization buffer and passed through a filter to perform flow cytometry (ACEA NovoCyte Quanteon, San Diego, CA, USA). Two xenografts per group were analyzed.

### 4.17. Statistical Analysis

Box plots were generated by RStudio. Fisher’s exact test was used for statistical analysis of mouse metastasis assays. The Chi-square test was used to examine the difference of cell composition for single-cell RNA sequencing data. The Student’s t-test was used for all other statistical analysis. The data were considered to be statistically significant with *p*-Value < 0.05.

### 4.18. Data Availability

WES data for an additional 12 cases generated in this study are available in SRA database with accession #SRP265671.

## 5. Conclusions

Our study is the first to demonstrate *CYLD* modulates fibroblast and endothelial cell infiltration in the NPC tumor microenvironment in addition to inhibiting tumorigenicity and metastasis. Our findings greatly enhance our understanding of the important role CYLD plays in NPC development via regulating NF-kB pathway. These findings provide promising directions for NPC therapies.

## Supplementary Materials

The following are available online at https://www.mdpi.com/2072-6694/12/7/1924/s1, Figure S1: HK1 cells were arrested in early S phase by twice thymidine treatment and release for 14 h, Figure S2: HONE-1-luc cell line with *CYLD* knockout/ control were intrasplenically injected into nude mice (ten mice per group), Figure S3: Wound healing assay was performed in HK1 cells, Figure S4: IF staining of p65 in HK1 and C17 confirm that p65 translocated into the nucleus from cytoplasm after *CYLD* KO with 30 min of 30 ng/mL TNF stimulation, Figure S5: IHC staining showed p65 expression and nucleus localization in C17 xenografts, Figure S6: QPCR screening of NF-kB downstream targets in HK1 and C17 cell lines and xenograft, Figure S7: NF-kB downstream targets were screened by QPCR in both HK1 xenografts with CYLD knockout/control, Figure S8: Mouse xenografts established from HK1 cells transduced with control and *CYLD* knockout were used for single cell RNA-Seq analysis, Figure S9: Knee plots and cell numbers captured in each experimental condition and other sequencing statistics, Figure S10: Full blot corresponding to Figure 1E. The protein ladder marker used in aa Western blots is shown on the right, Figure S11: Full blot corresponding to Figure 1F. Red box indicates the bands shown in the main figure, Figure S12: Full blot corresponding to Figure 4A. Red box indicates the bands shown in the figure, Figure S13: Full blot corresponding to Figure 4B. Red box indicates the bands shown in the figure, Figure S14: Full blot corresponding to Figure 7A. Red box indicates the bands shown in the figure, Figure S15: Full blot corresponding to Figure 7D. Red box indicates the bands shown in the figure, Table S1: Somatic alterations identified in *CYLD* from NPC, Table S2: *CYLD* mutations in the clinical specimens in NPC, Table S3: *CYLD* mutations in NPC cell lines, Table S4: List of primers used in real-time quantitative PCR, Table S5: List of cell markers for analysis of single cell RNA sequencing.
