# Supplementary material for: Cylindromatosis Lysine 63 Deubiquitinase (CYLD) Regulates NF-kB Signaling Pathway and Modulates Fibroblast and Endothelial Cells Recruitment in Nasopharyngeal Carcinoma
Source: Cancers (Basel). 2020 Jul 16;12(7):1924. doi: 10.3390/cancers12071924 (PMC7409113; doi:10.3390/cancers12071924)
Supplement: Supplementary file 1 [file cancers-12-01924-s001.pdf]

cancers

Supplementary Materials

Cylindromatosis Lysine 63 Deubiquitinase (CYLD) Regulates NF-kB Signaling Pathway and Modulates Fibroblast and Endothelial Cells Recruitment in Nasopharyngeal Carcinoma

Mingdan Deng, Wei Dai, Valen Zhuoyou Yu, Lihua Tao and Maria Li Lung

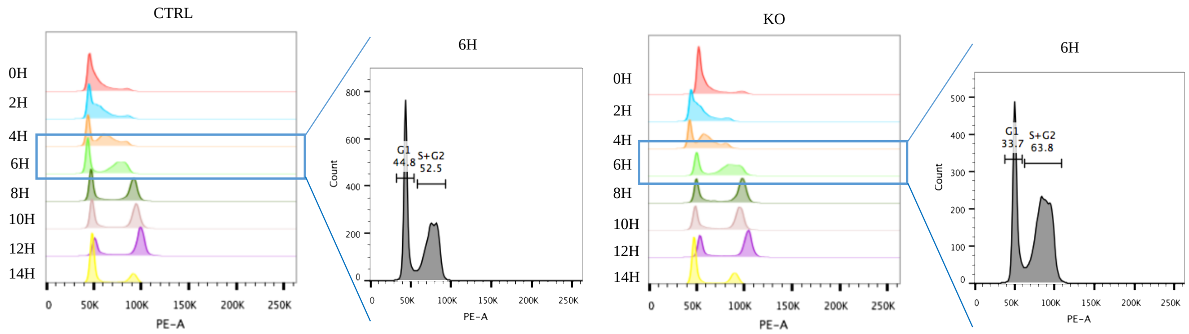

Figure S1. HK1 cells were arrested in early S phase by twice thymidine treatment and release for 14 h. Cells were collected every two hours and analyzed by flow cytometry. *CYLD* knockout HK1 cells transit from S to G2 phase faster than control cells at 6 h. CTRL: non-target control; KO: *CYLD*-target knockout.

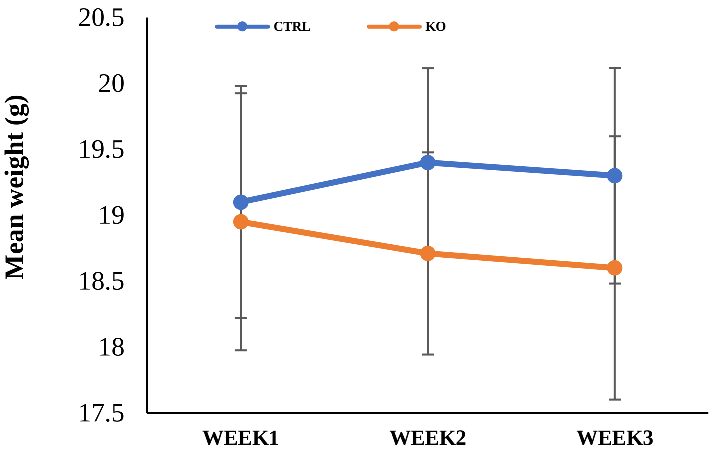

Figure S2. HONE-1-luc cell line with *CYLD* knockout/ control were intrasplenically injected into nude mice (ten mice per group). The body weight of each mouse was measured and recorded every week and is shown in the graph. CTRL: non-target control; KO: *CYLD*-target knockout.

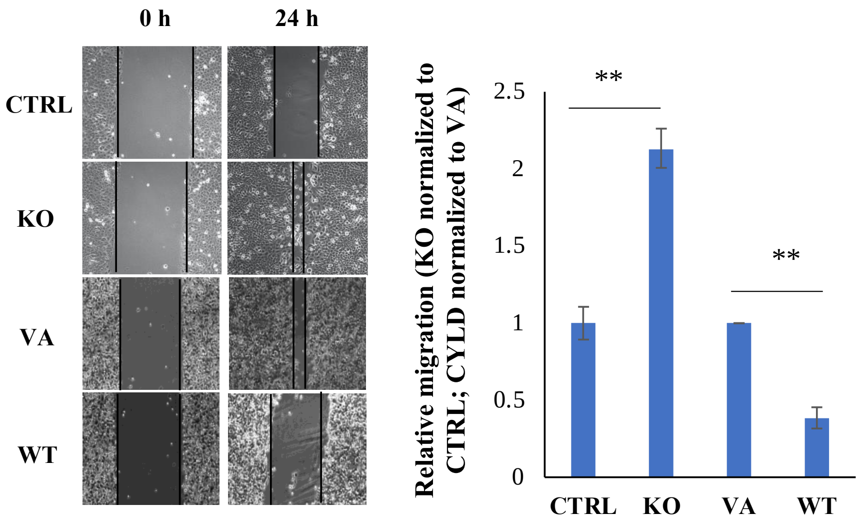

Figure S3. Wound healing assay was performed in HK1 cells. After 24 h of healing, the *CYLD* knockout group shows significantly faster migration compared to controls. The experiment was repeated three independent times. **: p-value < 0.01. CTRL: non-target control; KO: *CYLD*-target knockout; VA: overexpression vector-alone; WT: overexpression *CYLD* wild-type. Representative images are shown under 10× magnification.

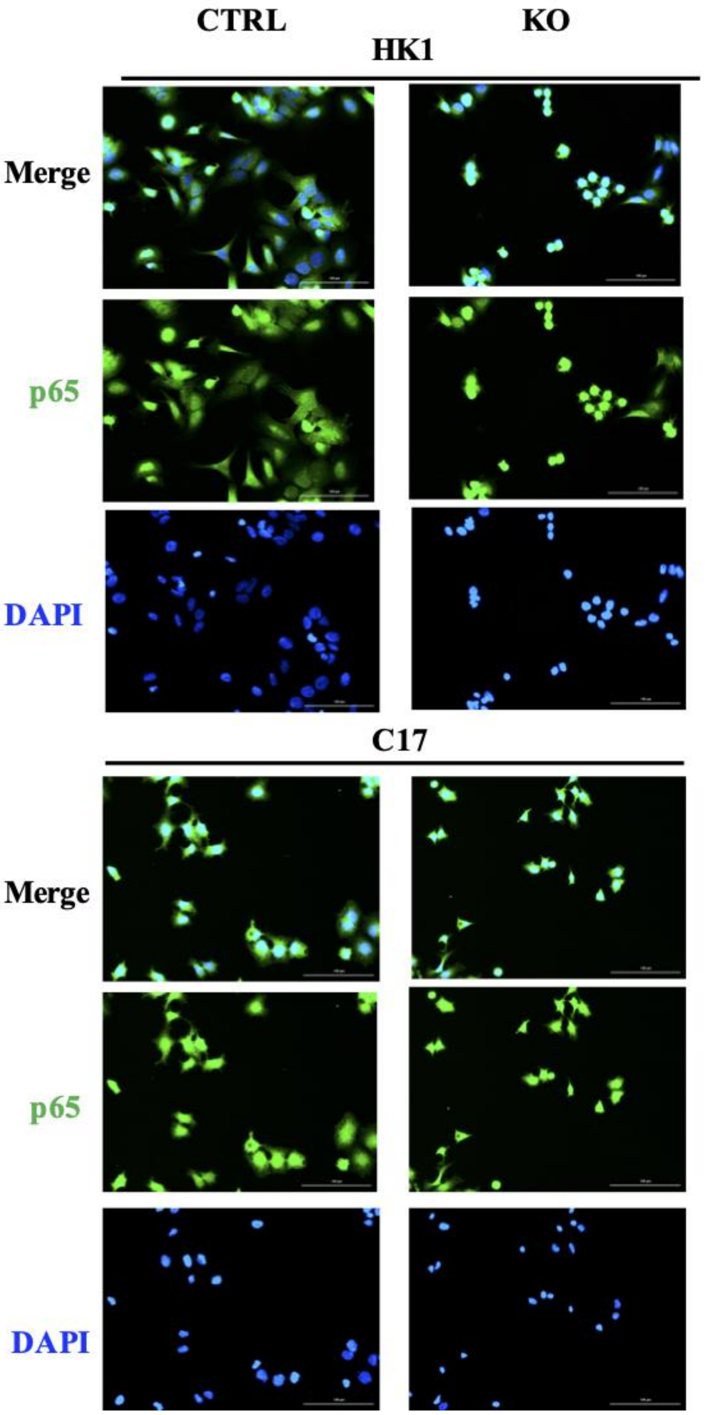

Figure S4. IF staining of p65 in HK1 and C17 confirm that p65 translocated into the nucleus from cytoplasm after *CYLD* KO with 30 min of 30 ng/mL TNF stimulation. CTRL: non-target control; KO: *CYLD*-target knockout. Scale Bar = 100 μm.

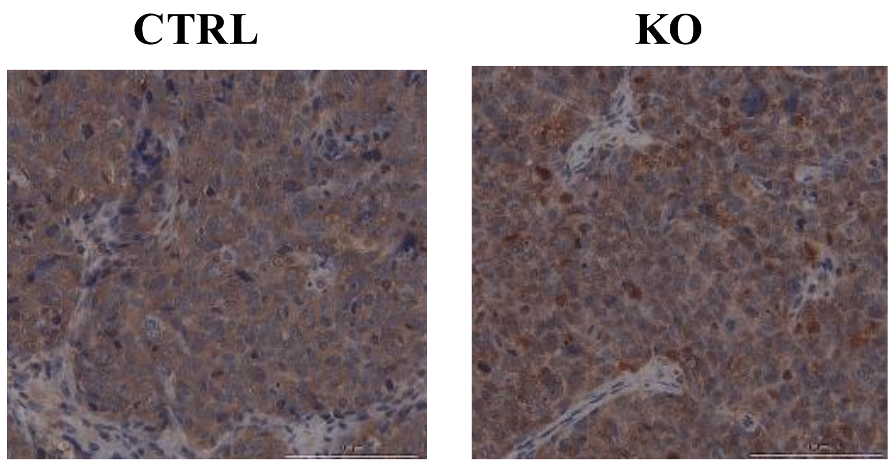

Figure S5. IHC staining showed p65 expression and nucleus localization in C17 xenografts. Images were captured under 20× magnification. The xenograft with knockout shows higher density of p65 and more p65 nucleus positive staining than the control. Scale bar = 100 µm. CTRL: non-target control; KO: *CYLD*-target knockout.

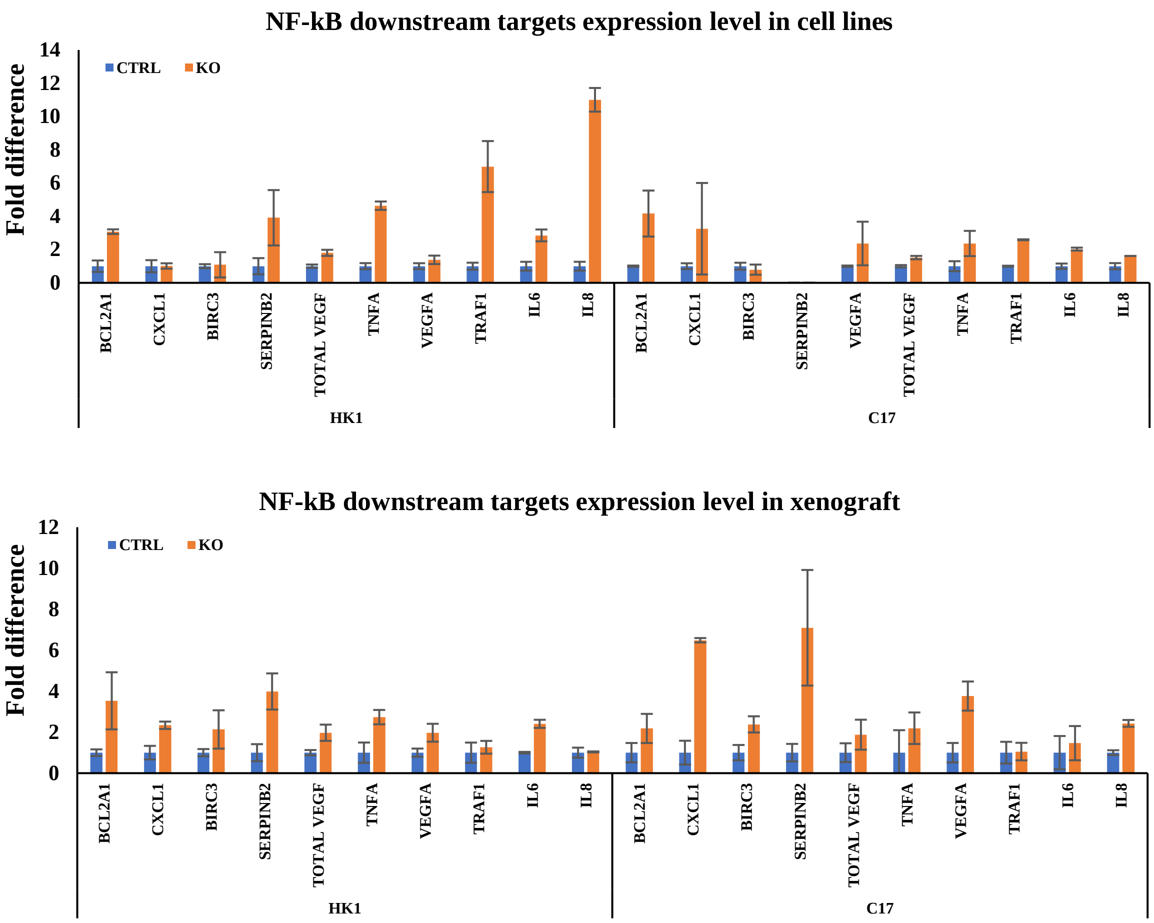

Figure 6S. QPCR screening of NF-kB downstream targets in HK1 and C17 cell lines and xenograft. CTRL: non-target control; KO: *CYLD*-target knockout.

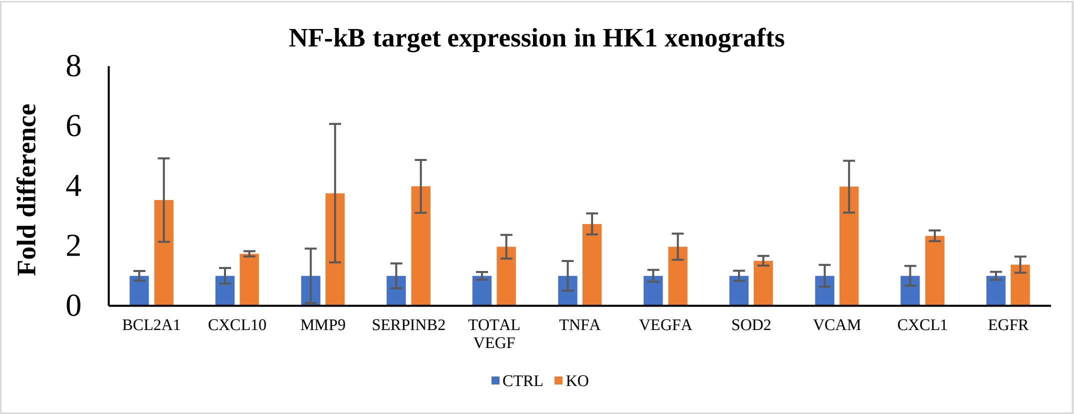

Figure S7. NF-kB downstream targets were screened by QPCR in both HK1 xenografts with CYLD knockout/control. NF-kB targets were upregulated after CYLD knockout, including *BCL2A1, CXCL10, MMP9, SERPINB2, total VEGF, TNFA, VEGFA, SOD2, VCAM, CXCL1 and EGFR*. CTRL: non-target control; KO: *CYLD*-target knockout. The data shown are represented as mean ± standard deviation (SD); n = 4.

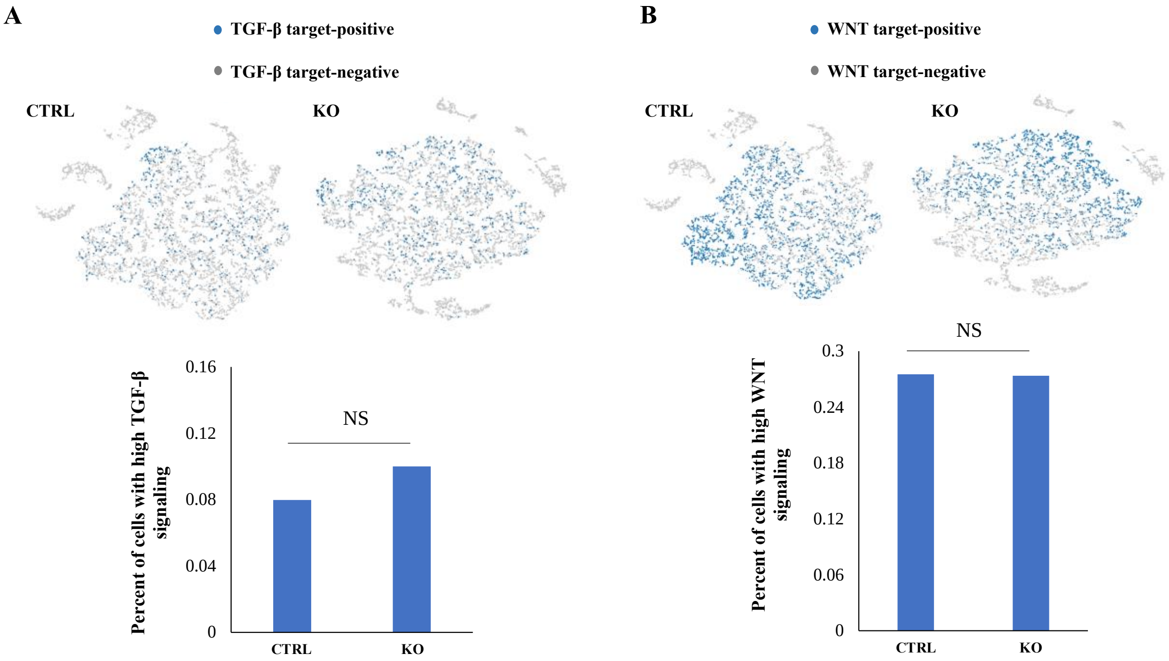

Figure S8. Mouse xenografts established from HK1 cells transduced with control and *CYLD* knockout were used for single cell RNA-Seq analysis. **A**) The panel of TGF-β downstream genes set as an indicator of high TGF-β activity in human cancer cells. These genes include *TGFB1, BMP2, GDF1, PDGFB, SNAIL1, SNAIL2, SOX4, LIF, HDM2, FOXP3*. The clusters show single cells with TGF-β target-positive (blue color)/target-negative (grey color). **B**) The panel of WNT downstream genes set as an indicator of high WNT activity in human cancer cells. These genes include *MYC, CCND1, CCN6, MMP7, BIRC5, DLL1, DKK1, FZD7, JUN*. The clusters show single cells with WNT target-positive (blue color)/target-negative (grey color). The bar graph shows the percent of high TGF-β/WNT signaling. CTRL: non-target control; KO: *CYLD*-target knockout. NS: not statistically significant.

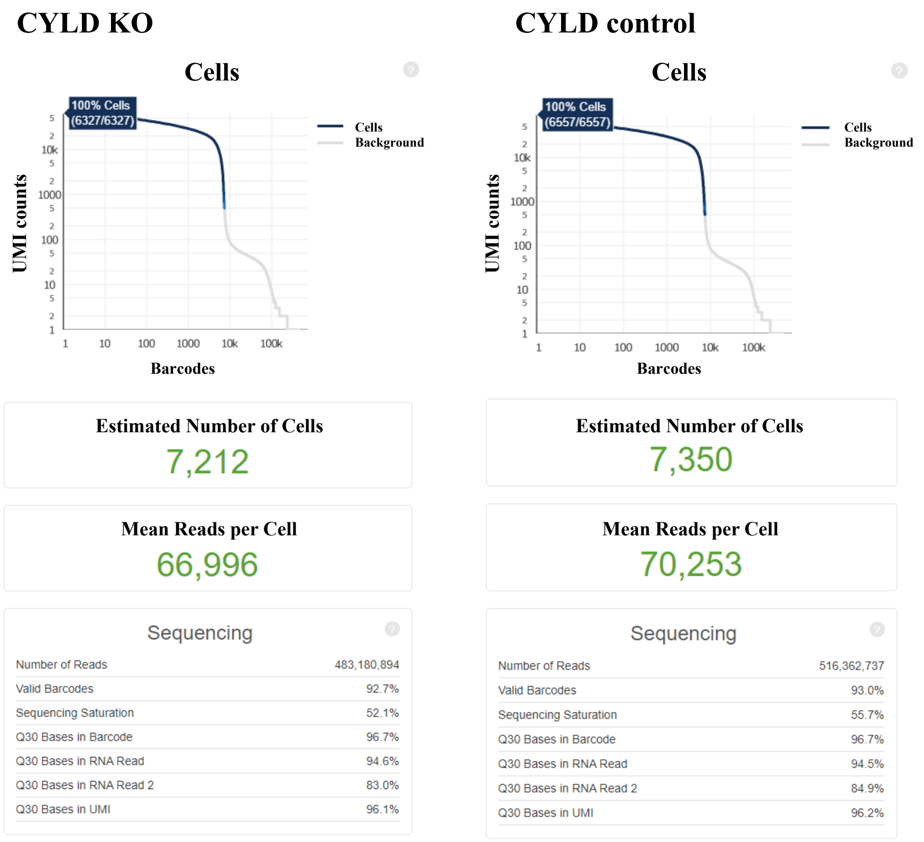

Figure S9. Knee plots and cell numbers captured in each experimental condition and other sequencing statistics.

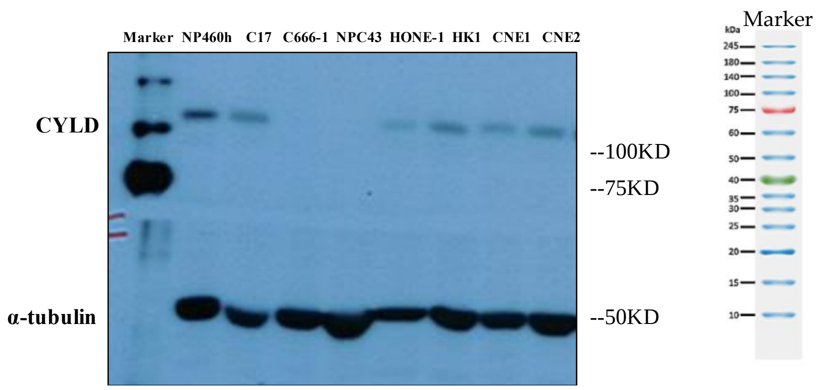

Figure S10. Full blot corresponding to Figure 1E. The protein ladder marker used in aa Western blots is shown on the right.

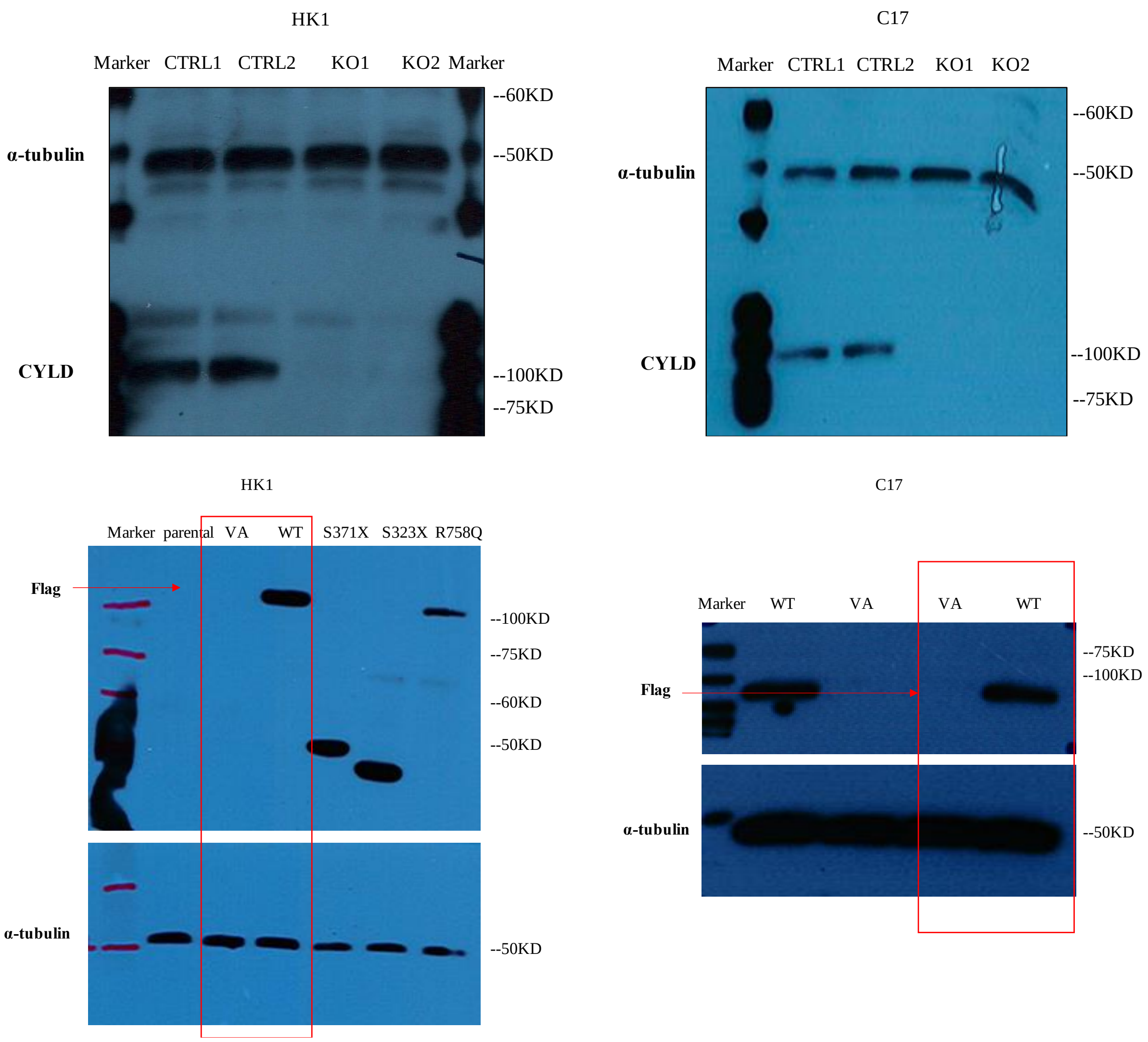

Figure S11. Full blot corresponding to Figure 1F. Red box indicates the bands shown in the main figure.

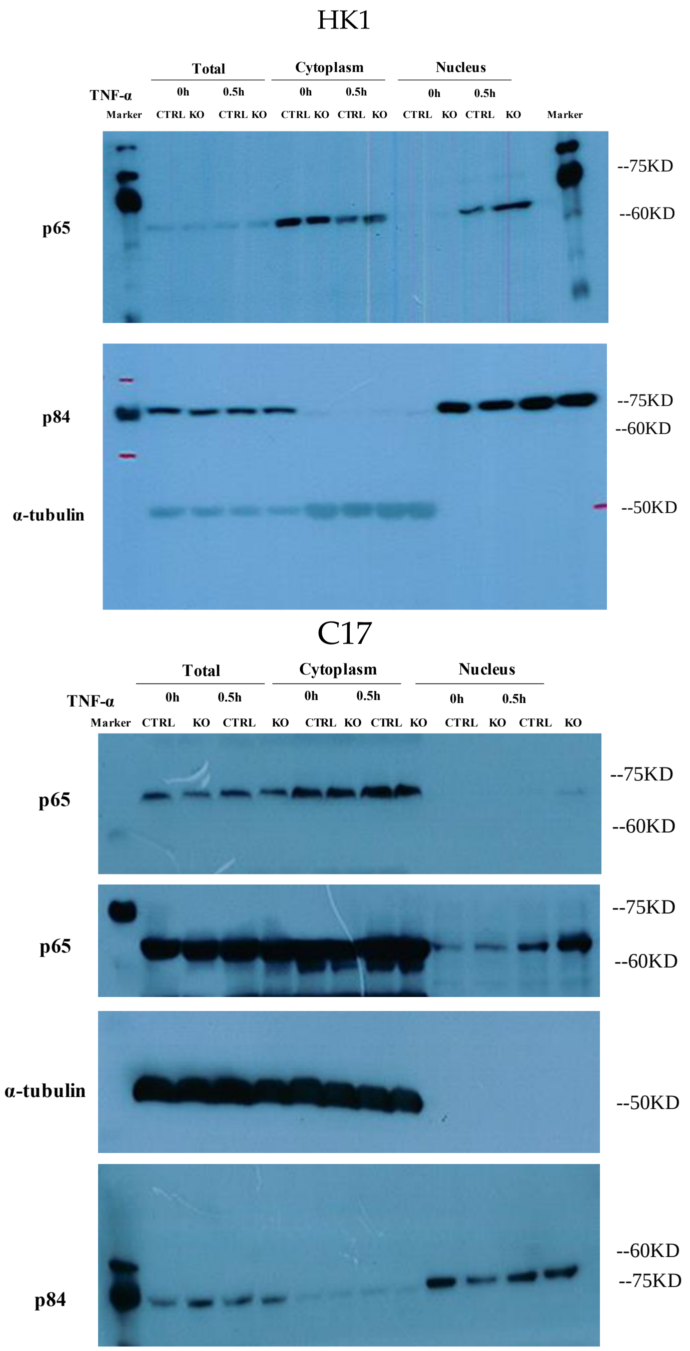

Figure S12. Full blot corresponding to Figure 4A. Red box indicates the bands shown in the figure.

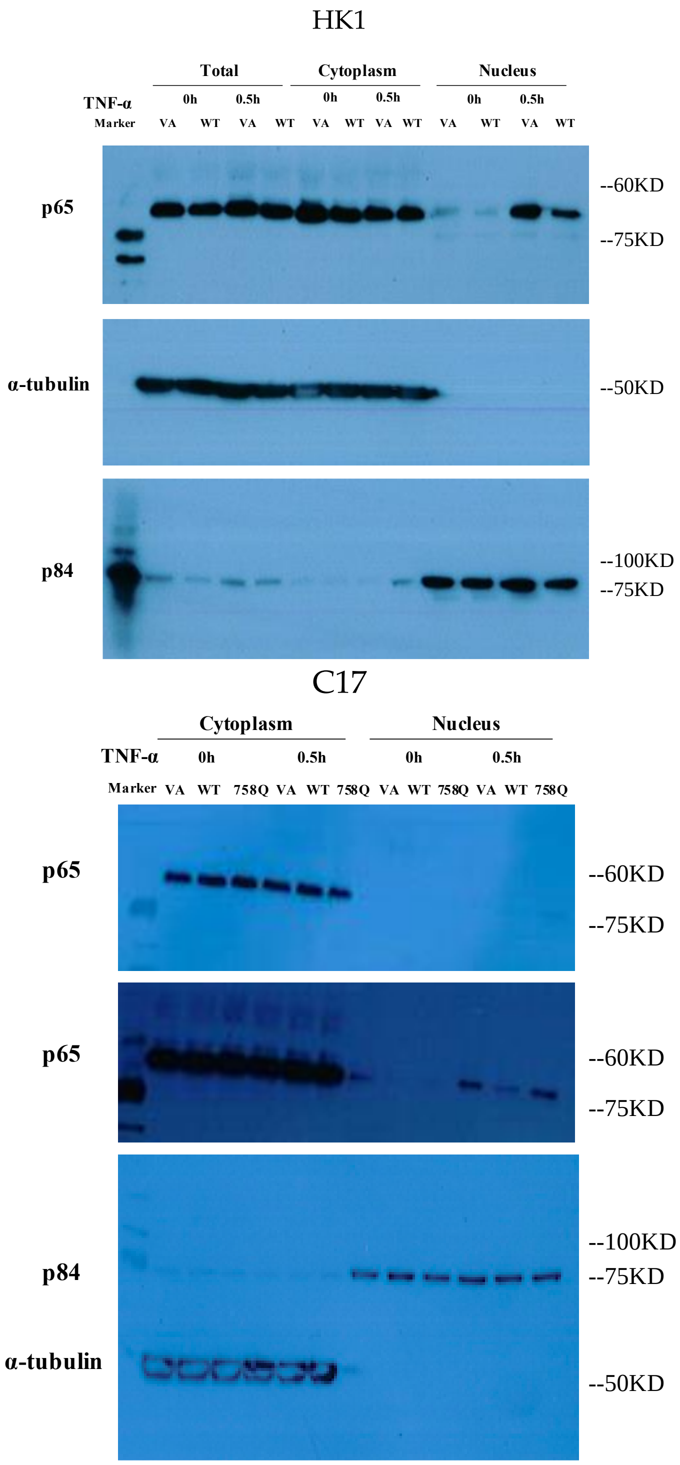

Figure S13. Full blot corresponding to Figure 4B. Red box indicates the bands shown in the figure.

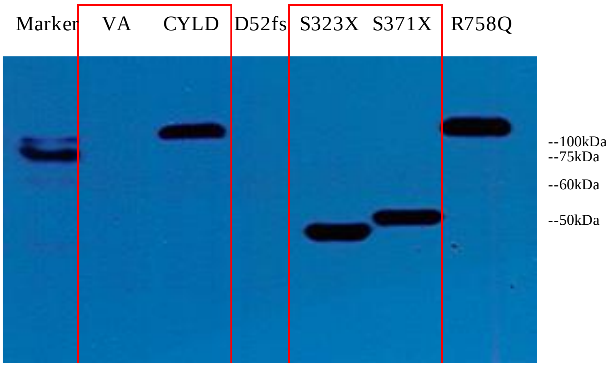

Figure S14. Full blot corresponding to Figure 7A. Red box indicates the bands shown in the figure.

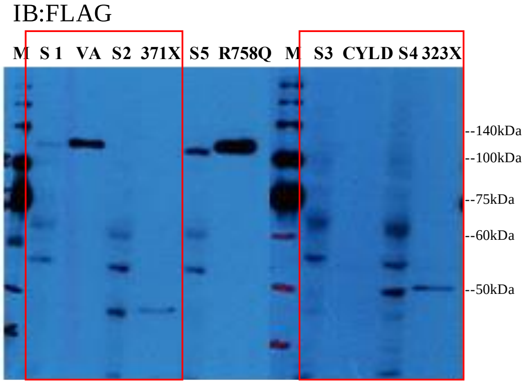

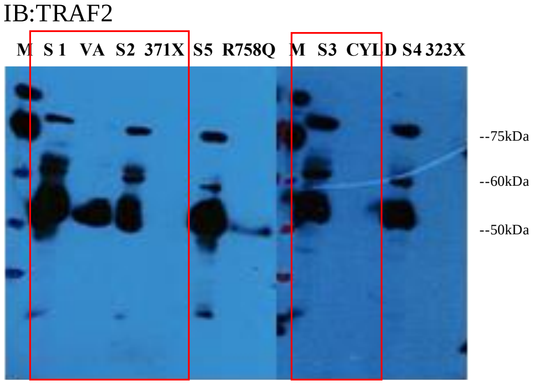

Figure S15. Full blot corresponding to Figure 7D. Red box indicates the bands shown in the figure.

Table S1. Somatic alterations identified in *CYLD* from NPC.

Sample	Reference	Gene	Alterations
NPCHKE14T	[1]	CYLD:NM_015247	Heterozygous deletion
NPCHKE22T	[1]	CYLD:NM_015247	Heterozygous deletion
NPCHKE30T	[1]	CYLD:NM_015247	Heterozygous deletion
NPCHKE40T	[1]	CYLD:NM_015247	Heterozygous deletion
CASE35T	[1]	CYLD:NM_015247	Heterozygous deletion
CASE21T	[1]	CYLD:NM_015247	Heterozygous deletion
CASE27T	[1]	CYLD:NM_015247	Heterozygous deletion
NPCHKE55T	#SRP265671	CYLD:NM_015247	Heterozygous deletion
NPCHKE56T	#SRP265671	CYLD:NM_015247	Heterozygous deletion
NPCHKE57T	#SRP265671	CYLD:NM_015247	Heterozygous deletion
NPC26FT	[2]	CYLD:NM_015247	Heterozygous deletion
NPC17FT	[2]	CYLD:NM_015247	Heterozygous deletion
HKNPC-085T	[3]	CYLD:NM_015247	Heterozygous deletion
HKNPC-046T	[3]	CYLD:NM_015247	Heterozygous deletion
HKNPC-090T	[3]	CYLD:NM_015247	Heterozygous deletion
HKNPC-066T	[3]	CYLD:NM_015247	Heterozygous deletion
HKNPC-052T	[3]	CYLD:NM_015247	Heterozygous deletion
HKNPC-101T	[3]	CYLD:NM_015247	Heterozygous deletion
HKNPC-024T	[3]	CYLD:NM_015247	Heterozygous deletion
HKNPC-072T	[3]	CYLD:NM_015247	Heterozygous deletion
HKNPC-084T	[3]	CYLD:NM_015247	Heterozygous deletion
HKNPC-094T	[3]	CYLD:NM_015247	Heterozygous deletion
HKNPC-050T	[3]	CYLD:NM_015247	Heterozygous deletion
HKNPC-096T	[3]	CYLD:NM_015247	Heterozygous deletion
HKNPC-075T	[3]	CYLD:NM_015247	Heterozygous deletion
HKNPC-037T	[3]	CYLD:NM_015247	Heterozygous deletion
HKNPC-071T	[3]	CYLD:NM_015247	Heterozygous deletion

HKNPC-065T	[3]	CYLD:NM_015247	Heterozygous deletion
HKNPC-033T	[3]	CYLD:NM_015247	Heterozygous deletion
HKNPC-003T	[3]	CYLD:NM_015247	Heterozygous deletion
HKNPC-078T	[3]	CYLD:NM_015247	Heterozygous deletion
HKNPC-097T	[3]	CYLD:NM_015247	Heterozygous deletion
HKNPC-036T	[3]	CYLD:NM_015247	Heterozygous deletion
HKNPC-011T	[3]	CYLD:NM_015247	Heterozygous deletion
HKNPC-016T	[3]	CYLD:NM_015247	Heterozygous deletion
HKNPC-008T	[3]	CYLD:NM_015247	Heterozygous deletion
HKNPC-004T	[3]	CYLD:NM_015247	Heterozygous deletion
HKNPC-005T	[3]	CYLD:NM_015247	Heterozygous deletion
HKNPC-088T	[3]	CYLD:NM_015247	Heterozygous deletion
HKNPC-076T	[3]	CYLD:NM_015247	Homozygous deletion
HKNPC-034T	[3]	CYLD:NM_015247	Heterozygous deletion/truncation E720fs
HKNPC-064T	[3]	CYLD:NM_015247	Heterozygous deletion/truncation S371X
HKNPC-079T	[3]	CYLD:NM_015247	Heterozygous deletion/truncation Q707X
HKNPC-087T	[3]	CYLD:NM_015247	Heterozygous deletion/missense S600F
HKNPC-099T	[3]	CYLD:NM_015247	Heterozygous deletion/truncation I769fs
HKNPC-049T	[3]	CYLD:NM_015247	Heterozygous deletion/tandem duplication [$]
HKNPC-098T	[3]	CYLD:NM_015247	Heterozygous deletion/missense H871N
HKNPC-043T	[3]	CYLD:NM_015247	Heterozygous deletion/truncation Q898X
HKNPC-006T	[3]	CYLD:NM_015247	Heterozygous deletion/truncation C526fs
HKNPC-007T	[3]	CYLD:NM_015247	Heterozygous deletion/truncation Q731X
NPCHKE4T	[1]	CYLD:NM_015247	Heterozygous deletion/D52fs
NPCHKE7T	[1]	CYLD:NM_015247	GAIN
NPCHKE11T	[1]	CYLD:NM_015247	GAIN
NPCHKE59T	#SRP265671	CYLD:NM_015247	GAIN
NPC10FT	[2]	CYLD:NM_015247	GAIN
NPC38FT	[2]	CYLD:NM_015247	GAIN
NPC9DT	[2]	CYLD:NM_015247	GAIN
NPC31FT	[2]	CYLD:NM_015247	GAIN
NPC9FT	[2]	CYLD:NM_015247	GAIN
HKNPC-021T	[3]	CYLD:NM_015247	GAIN
HKNPC-105T	[3]	CYLD:NM_015247	GAIN
HKNPC-060T	[3]	CYLD:NM_015247	GAIN
HKNPC-057T	[3]	CYLD:NM_015247	GAIN
HKNPC-061T	[3]	CYLD:NM_015247	GAIN
HKNPC-055T	[3]	CYLD:NM_015247	GAIN
HKNPC-022T	[3]	CYLD:NM_015247	GAIN
HKNPC-035T	[3]	CYLD:NM_015247	GAIN
HKNPC-014T	[3]	CYLD:NM_015247	GAIN
HKNPC-013T	[3]	CYLD:NM_015247	GAIN
HKNPC-010T	[3]	CYLD:NM_015247	Amplification
HKNPC-029T	[3]	CYLD:NM_015247	truncation S344X
HKNPC-063T	[3]	CYLD:NM_015247	truncation S371X
HKNPC-015T	[3]	CYLD:NM_015247	truncation F763fs
NPCHKE51T	[1]	CYLD:NM_015247	truncation S323X
NPCHKE50T	[1]	CYLD:NM_015247	truncation S371X

[$] Alteration was identified by whole genome sequencing in the previous genomics study.

Table S2. *CYLD* mutations in the clinical specimens in NPC.

Sample ID	Chr	Start	End	Reference Allele	Alternative Allele	Gene	Type	Amino Acid Change	CADD Score *	Cosmic Database	ClinVar Database
NPCHKE4T	chr16	50783765	50783772	TCGTTCTG	-	CYLD	Frameshift Deletion	D52fs	NA	NA	NA
NPCHKE51T	chr16	50810135	50810135	C	G	CYLD	Stopgain	S323X	40	NA	NA
HKNPC-029T	chr16	50811745	50811745	C	G	CYLD	Stopgain	S344X	37	ID=COSM3937084;OCCURENCE=1(oesophagus)	NA
HKNPC-063T	chr16	50811826	50811826	C	A	CYLD	Stopgain	S371X	38	ID=COSM43402;OCCURENCE=1(skin)	Pathogenic
HKNPC-064T	chr16	50811826	50811826	C	A	CYLD	Stopgain	S371X	38	ID=COSM43402;OCCURENCE=1(skin)	Pathogenic
NPCHKE50T	chr16	50811826	50811826	C	A	CYLD	Stopgain	S371X	38	ID=COSM43402;OCCURENCE=1(skin)	Pathogenic
HKNPC-006T	chr16	50815216	50815220	TGCCC	-	CYLD	Frameshift Deletion	C526fs	NA	NA	NA
HKNPC-087T	chr16	50816350	50816350	C	T	CYLD	Missense	S600F	26.3	NA	NA
HKNPC-079T	chr16	50825479	50825479	C	T	CYLD	Stopgain	Q707X	39	ID=COSM1666909;OCCURENCE=1(skin)	NA
HKNPC-034T	chr16	50825518	50825518	-	A	CYLD	Frameshift Insertion	E720fs	NA	NA	NA
HKNPC-007T	chr16	50825551	50825551	C	T	CYLD	Stopgain	Q731X	39	NA	NA
HKNPC-015T	chr16	50826553	50826553	T	-	CYLD	Frameshift Deletion	F763fs	NA	NA	NA
HKNPC-099T	chr16	50826571	50826571	-	T	CYLD	Frameshift Insertion	I769fs	NA	NA	NA
HKNPC-098T	chr16	50828264	50828264	C	A	CYLD	Missense	H871N	28.7	NA	NA
HKNPC-043T	chr16	50830240	50830240	C	T	CYLD	Stopgain	Q898X	38	NA	NA

* Combined Annotation Dependent Depletion score.

Table S3. *CYLD* mutations in NPC cell lines.

Sample ID	Chr	Start	End	Reference Allele	Alternative Allele	Gene	Type	Amino Acid Change	Clinical Characteristics
C666-1	chr16	50826571	50826571	-	T	CYLD	Frameshift Insertion *	I769fs	Hong Kong, male, poor differentiation [4]
NPC43	chr16	46503136	56362863			CYLD	Deletion $		Hong Kong, male, poor differentiation [5]
C17	-	-	-	-	-		No mutation *		France, male, poor differentiation [6]
HK1	-	-	-	-	-		No mutation *		Hong Kong, male, well differentiation [7]
HONE1	-	-	-	-	-	CYLD	unknown	-	Mainland China, male, poor differentiation [8]
CNE1	-	-	-	-	-	CYLD	unknown	-	Mainland China, male, well differentiation [9]
CNE2	-	-	-	-	-	CYLD	unknown	-	Mainland China, male, poor differentiation [10]
NP460hTert	-	-	-	-	-	CYLD	unknown	-	Hong Kong, male [11]

* Mutation was inferred from the RNA sequencing data [5]; $ Deletion was inferred from the WES data [5]

Table S4. List of primers used in real-time quantitative PCR.

Gene	Primer Sequence (5′to 3′)
Total VEGF-F	ACTGCCATCCAATCGAGACC
Total VEGF-R	GATGGCTTGAAGATGTACTCGATCT
BCL2L1-F	ACCACATCCTCCGTCCAG
BCL2L1-R	ACTAGCTGCAAGGGACCAGA
BIRC3-F	GATGAAAATGCAGAGTCATCAATTA
BIRC3-R	CATGATTGCATCTTCTGAATGG
CXCL10-F	GAAAGCAGTTAGCAAGGAAAGGT
CXCL10-R	GACATATACTCCATGTAGGGAAGTGA
BIRC5-F	CACCGCATCTCTACATTCAAGA
BIRC5-R	CAAGTCTGGCTCGTTCTCAGT
EGFR-F	GTGGCATTTAGGGGTGACTC
EGFR-R	TCAGAATATCCAGTTCCTGTGG
CXCL1-F	TCCTGCATCCCCCATAGTTA
CXCL1-R	CTTCAGGAACAGCCACCAGT
MMP9-F	GAACCAATCTCACCGACAGG
MMP9-R	GCCACCCGAGTGTAACCATA
SOD2-F	GCTCCGGTTTTGGGGTAT
SOD2-R	CCGTAGTCGTAGGGCAGGT
VEGFA-F	TCTTCAAGCCATCCTGTGTG
VEGFA-R	CAGTGGGCACACACTCCA
TNFA-F	ACTTTGGAGTGATCGGCC
TNFA-R	GCTTGAGGGTTTGCTACAAC

Table S5. List of cell markers for analysis of single cell RNA sequencing.

Cell Type	Gene
natural killer (NK) cell	*Nkg7, Ncr1, Gzma, Il18rap, S1pr5*
macrophage	*Cd68, Ms4a7, F4/80*
endothelial cell	*Cd31*
neutrophil	*Ly6g*
fibroblast	*Acta2, Myh11, Tagln, Colla2, Col3a1*

References.

1. Zheng, H.; Dai, W., Cheung, A.K.; Ko, J.M.; Kan, R.; Wong, B.W.; Leong, M.M.; Deng, M.; Kwok, T.C.; Chan, J.Y.; et al. Whole-exome sequencing identifies multiple loss-of-function mutations of NF-kappaB pathway regulators in nasopharyngeal carcinoma. *Proc. Natl. Acad. Sci. USA* **2016**, *113*, 11283–11288.
2. Lin, D.C.; Meng, X.; Hazawa, M.; Nagata, Y.; Varela, A.M.; Xu, L.; Sato, Y.; Liu, L.Z.; Ding, L.W.; Sharma, A.; et al. The genomic landscape of nasopharyngeal carcinoma. *Nat. Genet* **2014**; 46, 866–871.
3. Li, Y.Y.; Chung, G.T.; Lui, V.W.; To, K.F.; Ma, B.B.; Chow, C.; Woo, J.K.; Yip, K.Y.; Seo, J.; Hui, E.P.; et al. Exome and genome sequencing of nasopharynx cancer identifies NF-kappaB pathway activating mutations. *Nat. Commun.* **2017**, *8*, 14121.
4. Cheung, S.T.; Huang, D.P.; Hui, A.B.; Lo, K.W.; Ko, C.W.; Tsang, Y.S.; Wong, N.; Whitney, B.M.; Lee, J.C. Nasopharyngeal carcinoma cell line (C666-1) consistently harbouring Epstein-Barr virus. *Int. J. Cancer* **1999**, *83*, 121–126, doi:10.1002/(sici)1097-0215(19990924)83:13.0.co;2-f.

5. Lin, W.; Tsao, S.W.; Jia, L.; Deng, W.; Zheng, H.; Dai, W.; Ko, J.; Lo, K.W.; Chung, G.T.Y.; Yip, K.; et al. Establishment and characterization of new tumor xenografts and cancer cell lines from EBV-positive nasopharyngeal carcinoma. *Nat. Commun.* **2018**, *9*, 4663, doi:10.1038/s41467-018-06889-5.
6. Busson, P.; Ganem, G.; Flore, P.; Mugneret, F.; Clausse, B.; Caillou, B.; Braham, H.; Wakasugi, H.; Lipinski, M.; Tursz, T. Establishment and characterization of three transplantable EBV-containing nasopharyngeal carcinomas. *Int. J. Cancer* **1988**, *42*, 599–606.
7. Huang, D.P.; Ho, J.H.; Poon, Y.F.; Chew, E.C.; Saw, D.; Lui, M.; Li, C.L.; Mak, L.S.; Lai, S.H.; Lau, W.H. Establishment of a cell line (NPC/HK1) from a differentiated squamous carcinoma of the nasopharynx. *Int. J. Cancer* **1980**, *26*, 127–132.
8. Yao, K.T.; Zhang, H.Y.; Zhu, H.C.; Wang, F.X.; Li, G.Y.; Wen, D.S.; Li, Y.P.; Tsai, C.H.; Glaser, R. Establishment and characterization of two epithelial tumor cell lines (HNE-1 and HONE-1) latently infected with Epstein-Barr virus and derived from nasopharyngeal carcinomas. *Int. J. Cancer* **1990**, *45*, 83–89.
9. Zeng, Y. Establishment of an epitheloid cell line and a fusiform cell line from a patient with nasopharyngeal carcinoma. *Sci. Sin.* **1978**, 21, 127–134.
10. Gu, S.U.; Tann, B.F.; Zeng, Y.; Zhou, W.P.; Li, K.; Zhao, M.C. Establishment of an epithelial cell line (CNE-2) from an NPC patient with poorly differentiated squamous cell carcinoma. *Chin. J. Cancer* **1983**, *2*, 70–72.
11. Tsao, S.W.; Wang, X.; Liu, Y.; Cheung, Y.C.; Feng, H.C.; Zheng, Z.; Wong, N.; Yuen, P.W.; Lo, K.F.; Wong, Y.C.; et al. Establishment of two immortalized nasopharyngeal epithelial cell lines using SV40 large T and HPV16E6/E7 viral oncogenes. *Biochim. Biophys. Acta* **2002**, *1590*, 150–158.
